# Interplay of Chimeric Mating-Type Loci Impairs Fertility Rescue and Accounts for Intra-Strain Variability in *Zygosaccharomyces rouxii* Interspecies Hybrid ATCC42981

**DOI:** 10.3389/fgene.2019.00137

**Published:** 2019-03-01

**Authors:** Melissa Bizzarri, Stefano Cassanelli, Laura Bartolini, Leszek P. Pryszcz, Michala Dušková, Hana Sychrová, Lisa Solieri

**Affiliations:** ^1^Department of Life Sciences, University of Modena and Reggio Emilia, Reggio Emilia, Italy; ^2^Laboratory of Zebrafish Developmental Genomics, International Institute of Molecular and Cell Biology, Warsaw, Poland; ^3^Department of Membrane Transport, Institute of Physiology, Czech Academy of Sciences, Prague, Czechia

**Keywords:** mating-type, MinION, sexual cycle, *Zygosaccharomyces*, chimeric loci, interspecies hybridization, yeast

## Abstract

The pre-whole genome duplication (WGD) *Zygosaccharomyces* clade comprises several allodiploid strain/species with industrially interesting traits. The salt-tolerant yeast ATCC42981 is a sterile and allodiploid strain which contains two subgenomes, one of them resembling the haploid parental species *Z. rouxii*. Recently, different mating-type-like (*MTL*) loci repertoires were reported for ATCC42981 and the Japanese strain JCM22060, which are considered two stocks of the same strain. *MTL* reconstruction by direct sequencing approach is challenging due to gene redundancy, structure complexities, and allodiploid nature of ATCC42981. Here, DBG2OLC and MaSuRCA hybrid *de novo* assemblies of ONT and Illumina reads were combined with *in vitro* long PCR to definitively solve these incongruences. ATCC42981 exhibits several chimeric *MTL* loci resulting from reciprocal translocation between parental haplotypes and retains two *MAT*a/*MAT*α expression loci, in contrast to *MAT*α in JCM22060. Consistently to these reconstructions, JCM22060, but not ATCC42981, undergoes mating and meiosis. To ascertain whether the damage of one allele at the *MAT* locus regains the complete sexual cycle in ATCC42981, we removed the *MAT*α expressed locus by gene deletion. The resulting *MAT*a/- hemizygous mutants did not show any evidence of sporulation, as well as of self- and out-crossing fertility, probably because incomplete silencing at the chimeric *HML*α cassette masks the loss of heterozygosity at the *MAT* locus. We also found that *MAT*α deletion switched off a2 transcription, an activator of a-specific genes in pre-WGD species. These findings suggest that regulatory scheme of cell identity needs to be further investigated in *Z. rouxii* protoploid yeast.

## Introduction

Polyploidization, a state resulting from doubling of a genome within a species (autopolyploidy) or the merging between different species (allopolyploidy) (Campbell et al., 2016), is an important evolutionary force which shapes eukaryotic genomes (Albertin and Marullo, 2012), triggers speciation, and can result in phenotypic changes driving adaptation (Ohno, 1970). A whole-genome duplication (WGD) event occurred approximately 100–200 Mya in the common ancestor of six yeast genera in the family Saccharomycetaceae, including *Saccharomyces cerevisiae* (as reviewed by Wolfe et al., 2015). WGD was recently proposed to be a direct consequence of an ancient hybridization between two ancestral species (Marcet-Houben and Gabaldón, 2015), followed by genome doubling of initially sterile hybrid to regain fertility, *i.e.*, the ability to undergo meiosis and produce viable spore (Wolfe, 2015).

Different mechanisms can contribute to hybrid infertility, such as chromosomal missegregation caused by meiosis I non-disjunction (Boynton et al., 2018), chromosomal rearrangements (Liti et al., 2006; Rajeh et al., 2018), and Dobzhansky–Muller gene incompatibilities either between nuclear genes (Bizzarri et al., 2016) or between mitochondrial and nuclear genes (Lee et al., 2008). Specialized loci, called the mating-type (*MAT*)-like (*MTL*) cassettes, regulate mating between haploid cells with opposite *MAT*a and *MAT*α idiomorphs, as well as meiosis in diploid a/α cells. In diplontic yeast *S. cerevisiae MAT* locus on chromosome III contains either the a1 or the α1 and α2 genes in Ya and Yα segments, respectively, surrounded by X and Z regions at the left and right sides. In haploid α cells, α1 activates the α-specific genes (αsgs), while α2 represses a cohort of a-specific genes (asgs), which a cells transcribe by default (Haber, 2012). Finally, diploid a/α cells are meiosis but not mating-competent, because the a1-α2 heterodimer positively regulates *IME1* (Inducer of Meiosis) gene expression and represses the transcription of *RME1*, a haploid-specific gene (hsg) that inhibits entry into meiosis, and of other hsgs required for mating responses. *S. cerevisiae* cells also have extra copies of *MAT* genes at the *HMR*a and *HML*α loci located close to telomeres of chromosome III and silenced by a combination of the Sir1–4 proteins (Hickman et al., 2011). These extra copies serve as donors during the mating-type switching which enables *MAT*a cells to convert into *MAT*α cells, or vice versa, and to mate each other. This autodiploidization event is triggered by a site-specific endonuclease called HO which induces double-strand break at Z region of the *MAT* locus. In *Saccharomyces* interspecies hybrids, experimental deletion of one *MAT* locus or elimination of the entire chromosome carrying one *MAT* locus yielded fertile allotetraploids (Greig et al., 2002; Pfliegler et al., 2012; Karanyicz et al., 2017). More recently, the *MAT* locus damage was proposed to be the most plausible evolutionary route which enables natural interspecies hybrids of the *Zygosaccharomyces bailii* complex to rescue mating and meiosis (Ortiz-Merino et al., 2017; Braun-Galleani et al., 2018).

In the Saccharomycetaceae lineage, *Z. rouxii* stands on the crossroad where different and relevant evolutionary events take their way (Dujon and Louis, 2017). This evolutionary route involves ancient allopolyploidization between two parental lineages, one of which was close to *Z. rouxii* and *Torulaspora delbrueckii* (ZT) clade (Marcet-Houben and Gabaldón, 2015). *Z. rouxii* represents the early branching species before WGD that recruits HO from a LAGLIDADG intein to catalyze the first step of mating-type switching (Fabre et al., 2005). Furthermore, *Z. rouxii* exhibits the triplication of *MTL* loci, which is a genomic landmark of the Saccharomycetaceae family, but, in contrast to *S. cerevisiae*, it lacks of *MAT*-*HMR* linkage. Whereas the route of αsg regulation appears to be conserved, the regulatory circuit of asgs has been extensively rewired across the Saccharomycotina clade. Instead of the negative regulatory circuit widespread in post-WGD species, several pre-WGD species activate asgs by an HMG-domain protein (a2) that is encoded by *MAT*a (Tsong et al., 2003). Conventionally, *Z. rouxii* displays haplontic life style, where heterothallic haploid cells with opposite mating-type mate each other or, alternatively, homothallic haploid cells switch mating-type and subsequently undergo mating between mother and daughter cells. In both cases, the transient diploid zygote should sporulate to restore the haploid state. Alternatively, stable allodiploid strains arose from mating between divergent haploid parents. One parental haplotype (called T-subgenome) resembles *Z. rouxii* and was 15% different from the other parental haplotype (called P-subgenome) (Gordon and Wolfe, 2008; Bizzarri et al., 2016, 2018; Watanabe et al., 2017).

Both haploid and allodiploid strains show highly variable gene arrangements around *MTL*, suggesting that these loci are recombination hotspot during error-prone mating-type switching events (Watanabe et al., 2013; Solieri et al., 2014). Structural rearrangements are so rampant in these regions that different stock cultures of the same haploid (Watanabe et al., 2013) or allodiploid (Bizzarri et al., 2016; Watanabe et al., 2017) strains can display distinct *MTL* repertoires. For instance, differences in *MTL* loci were recently found between two sub-cultures of the allodiploid strain ATCC42981. In our previous work, we found 7 *MTL* loci in in-house stock of ATCC42981 (termed ATCC42981_R for convenience) (Bizzarri et al., 2016), while Watanabe et al. (2017) detected 6 *MTL* loci in strain JCM22060, the Japanese stock of ATCC42981. Ectopic recombination between *MTL*-flanking regions from divergent parental haplotypes yields chimeric arrangements hardly to resolve both by targeted long PCR approaches (Bizzarri et al., 2016) and by genome sequencing technologies based on short reads (Watanabe et al., 2017).

In 2014, the MinION sequencing device (Oxford Nanopore Technology, ONT) was released and initially exploited to sequence and assemble PCR products or microbial genomes (Jain et al., 2016). Recent improvements in protein pore (a laboratory-evolved *Escherichia coli* CsgG mutant named R9.4), library preparation techniques (1D ligation and 1D rapid), sequencing speed (450 bases/s), and control software enabled the usage of Nanopore sequence data, in combination with other sequencing technologies, for assembling eukaryotic genomes including yeasts, nematodes and human (Istace et al., 2017; Jansen et al., 2017; Yue et al., 2017; Jain et al., 2018). The main advantage of ONT is that reads can reach tens of kilobases (Jain et al., 2016), making more easy to resolve repeat regions and to detect structural variation. Recently, the genome of allodiploid strain ATCC42981_R was sequenced and assembled through a *de novo* hybrid strategy which combined MinION long and Illumina short reads (Bizzarri et al., 2018).

Here, we took advantage from the newly released genome of ATCC42981_R (Bizzarri et al., 2018), in order to resolve incongruences in the highly dynamic *MTL* loci. Furthermore, we deleted the expressed *MAT*α^P^ locus in ATCC42981_R to test whether the loss of *MAT* heterozygosity can induce genome doubling and rescue fertility in allodiploid cells of the ZT clade.

## Materials and Methods

### Strains, Plasmids, and Culture Conditions

Yeast strains and plasmids used in this study are listed in Table 1. Yeast cells were routinely propagated at 28°C in YPD (1% yeast extract, 2% peptone, 2% glucose) medium with 1.5% agar when necessary. Stock cultures were stored at -80°C with glycerol at final concentration of 25% (v/v) for long-term preservation. For sporulation and mating assays, MEA (5% malt extract, 2% agar) with and without 6% NaCl and YM (0.3% yeast extract, 0.5% peptone, 0.3% malt extract, 1% dextrose, 1.5% agar) media were used. *Z. parabailii* strain G21C was used as control for conjugated asci formation after growth on MEA medium. When required, YPD medium was supplemented with G418 (100 mg mL^-1^; MP Biomedicals, Germany) to the final concentration of 200 μg mL^-1^.

**Table 1:** Yeast strains and plasmid used in this work.

Strains	Other codes	Relevant characteristics	Reference
***Z. parabailii*** G21C	Na	Isolated from balsamic glaze	This work
***Z. rouxii* strains**			
ATCC42981_R	JCM22060	*MAT*a^T^*/MAT*α^P^	Solieri et al., 2008; Bizzarri et al., 2016, 2017
CBS4837	NCYC1682; NBRC1876	*MAT*α^P^	Solieri et al., 2008; Sato et al., 2017; Watanabe et al., 2017
CBS4838	NBRC1877	*MAT*a^P^	Solieri et al., 2008; Watanabe et al., 2017
**Transformants**			
ATCC42981 *MAT*αΔ clone_6	Na	*MAT*α^P^ *Δ::loxP-KanMX-loxP*; *MAT*a^T^	This work
ATCC42981 *MAT*αΔ clone_65	Na	*MAT*α^P^ *Δ::loxP-KanMX-loxP*; *MAT*a^T^	This work
ATCC42981 *MAT*αΔ clone_74	Na	*MAT*α^P^ *Δ::loxP-KanMX-loxP*; *MAT*a^T^	This work
ATCC42981 *MAT*αΔ clone_177	Na	*MAT*α^P^ *Δ::loxP-KanMX-loxP*; *MAT*a^T^	This work
**Plasmids**			
pUG6		*loxP-KanMX-loxP* module	Güldener et al., 1996

*ATCC42981_R represents in-house stock culture of strain ATCC42981. Other codes indicate the name of strains in other culture collections. Genotype reports Y sequence from the putatively expression active mating-type locus (MAT). T and P superscripts indicate Ya or Yα sequences from T- or P-subgenomes, respectively. Na, not available.*

### DNA Manipulations

DNA manipulations were performed according to standard protocols (Sambrook et al., 1989). Genomic DNA from yeast cells was isolated according to Hoffman and Winston (1987), while plasmid DNA from *E. coli* was isolated using the GenElute^TM^ Plasmid Miniprep Kit (Sigma). DNA quantity and quality were evaluated electrophoretically and spectrophotometrically using a NanoDrop ND-1000 device (Thermo Scientific, Waltham, MA, United States). Zymoclean^TM^ Gel DNA Recovery and DNA Clean & Concentrator^TM^-5 Kits (Zymo Research, Orange, CA, United States) were used for the isolation of DNA fragments from agarose gels and for PCR amplicons purification, respectively. Long PCR amplifications were carried out with rTAQ DNA polymerase (Takara Bio, Shiga, Japan) according to manufacturer’s instructions. For colony PCR 1 μl of DNA extracted with lithium acetate-SDS method (Lõoke et al., 2011) was amplified with DreamTaq polymerase (Thermo Scientific, Waltham, MA, United States) according to the manufacturer’s instructions in 20 μl reaction volume. All PCR amplifications were carried out in a T100 Thermal cycler (Bio-Rad, Hercules, CA, United States). All primers used in this study are listed in Supplementary Table S1.

### Genome Re-assembly

Hybrid assembly of ATCC42981_R genome from Oxford Nanopore and Illumina reads was released to the European Nucleotide Archive under accession number PRJEB26771 (Bizzarri et al., 2018). In the deposited assembly Platanus contigs were scaffolded into 33 scaffolds with corrected MinION reads using DBG2OLC (Ye et al., 2016). These scaffolds were submitted to two-step polishing with long reads using Racon v1.2.0 (Vaser et al., 2017) and with short reads using Pilon v1.22 (Walker et al., 2014), and, finally, reduced using Redundans v.014 (Pryszcz and Gabaldón, 2016). Here, both long and short reads were assembled jointly with the alternative assembly algorithm Maryland Super-Read Celera Assembler v.3.2.2 (MaSuRCA) (Zimin et al., 2017) with default settings. Gene identification and annotation were carried out through the Yeast Genome Annotation Pipeline (YGAP)^1^ without frameshift correction (Proux-Wéra et al., 2012). MaSuRCA assembly completeness was assessed by Benchmarking Universal Single-Copy Orthologs (BUSCO) v3.0.2 (Simão et al., 2015) using saccharomycetales_odb9 data set.

### *MTL* Loci Search and Sanger-Based Validation

Search for *MTL* loci on scaffolds generated by DBG2OLC and MaSuRCA hybrid assemblies was carried out with a custom BLAST server built using the Sequenceserver software package (Priyam et al., 2015). Ya and Yα sequences and *MTL* flanking genes from the haploid reference genome of *Z. rouxii* CBS732^T^ (Souciet et al., 2009) were used as queries.

The *in silico MTL* arrangements were *in vitro* validated by PCR and Sanger sequencing. Specific primer sets were built on *MTL*-flanking regions outside the X and Z regions (Supplementary Table S1). For putatively active *MAT*α^P^ cassette, walking strategy was adopted to cover ∼1 Kb downstream and upstream Yα (Wang et al., 2011). According Watanabe et al. (2017), *MTL* and flanking genes were marked with T and P superscripts when they shared >99% identity with *Z. rouxii* CBS732^T^ or with P-subgenome from allodiploid NBRC110957 (Watanabe et al., 2017), respectively. N superscript was used to identify gene variants divergent from both T and P counterparts (identity lower than 99%). The 5′ *MTL*-flanking gene ZYRO0F18524g was named as *CHA1*_L_ for brevity. Sequences were aligned with Clustal Omega (Sievers and Higgins, 2014) and viewed using Jalview (Waterhouse et al., 2009). Neighbor-joining (NJ) tree was built using MEGA v.6 software (Tamura et al., 2013).

### Deletion Cassettes Construction and Yeast Transformation

Deletion of the active *MAT*α locus from P-subgenome (abbreviated as *MAT*α^P^) was performed with the reusable *loxP-kanMX*-*loxP* cassette as described previously (Güldener et al., 1996). The MATα1/2cp2-kanMX-F-80nt and MATα1/2cp2-kanMX-R-80nt primers contained ∼80 bp homology sequences outside the X ad Z regions of *MAT*α^P^ locus, respectively, and were used to amplify the *kanMX* deletion cassette from pUG6. After purification, the resulting PCR product was used to transform *Z. rouxii* cells by electroporation with a modified protocol from Pribylova and Sychrova (2003). Briefly, cells were grown (28°C; 180 rpm) in 80 ml of YPD medium supplemented with 300 mM NaCl until the exponential phase (corresponding to OD_600nm_ of 0.7–0.8). After washing with ddH_2_O, cells were resuspended into 16 ml of TE buffer (Tris-hydrochloride buffer, pH 8.0, containing 1.0 mM EDTA) supplemented with 25 mM dithiothreitol and 20 mM LiAc, and incubated at 30°C for 30 min with gently shaking. Cells were centrifuged at 6,000 *g* for 5 min at 4°C, and washed twice by resuspension in 20 mL of ice-cold 1 M sorbitol. Finally, cells were washed in 5 ml of ice-cold 1M sorbitol and resuspended in 800 μl of ice-cold 1 M sorbitol. One hundred microliter of competent cell suspension were transferred into a pre-chilled 2-mm electroporation cuvette (Molecular Bioproducts Inc., San Diego, CA, United States) and 1 μg of *loxP*-*kanMX*-*loxP* deletion cassette was added before the electroporation at 2250 V/cm for 5 ms (Eporator, Eppendorf, Germany). Immediately after electroporation, 100 μl of ice-cold 1 M sorbitol were added to electroporation mixture. Before plating on selective YPDA medium supplemented with G418, the transformation mixtures were incubated for 2 h in 5 ml of YPD at 30°C. In G418^R^ clones, targeted gene disruption was confirmed by full-length, 5′-, and 3′-end diagnostic PCRs (Supplementary Figure S1).

### RNA Extraction, cDNA Synthesis and RT-PCR

RNA was extracted from ATCC42981 wild type and deletion mutants cultured in YPD and harvested at stationary phase, as previously reported (Solieri et al., 2016). RNAs were reverse transcribed using 0.5 μM oligo (dT) and RevertAid H Minus Reverse Transcriptase (Thermo Scientific, Waltham, MA, United States) according to the manufacturer’s instructions. cDNAs (25 ng) were amplified using DreamTaq polymerase with primers specific for different variants of *MAT*a1, *MAT*α1, and *MAT*α2 genes, as well as for T and P variants of asgs *AGA2, STE2*, and *STE6* (Supplementary Table S1).

## Results

### Inventory of ATCC42981_R *MTL* Cassettes

To unambiguously characterize *MTL* loci in our stock culture, we exploited the new available ATCC42981_R draft genome (Bizzarri et al., 2018). This draft genome relies on the hybrid DBG2OLC assembly of MinION ultra-long and Illumina MiSeq short reads to resolve high heterozygosity and span repetitive regions, which represent the greatest technical challenges during the assembly of complex non-haploid genomes (Treangen and Salzberg, 2012; Del Angel et al., 2018).

Custom BLAST searches using Sequenceserver identified six scaffolds harboring 8 *MTL* loci (2 *MTL*α^T^, 4 *MTL*α^P^, and 2 *MTL*a) mainly at the scaffold edge (Table 2). As this pattern matched only partially either with our previous results (Bizzarri et al., 2016) or with the JCM22060 set of *MTL* loci (Watanabe et al., 2017), we took into account the possibility of misassembled segments, mainly considering that reference P-type genome is not available. Misassemblies could be more burdensome at the *MTL* loci which contain the long non-tandem repeated X and Z sequences enriched in homopolymeric stretches. To circumvent these caveats, we validated the *MTL* cassettes found in DBG2OLC assembly *in silico* by using the alternative assembler MaSuRCA, as well as *in vitro* by direct PCR and Sanger sequencing. With appropriate caution, agreement between these assemblies – which are completely independent in assembly algorithms – and among assemblies and Sanger sequencing can confirm the integrity of *MTL* cassettes.

**Table 2:** Overview of the *MTL* cassettes confirmed by hybrid *de novo* genome assemblies and PCR approach.

Cassette	DBG2OLC Scaffolds	Coordinates	PCR	MaSuRCA	JCM22060
**Yα^T^**					
*DIC1*^P^-*MTL*α^T^-ZYRO0F18634g^T^	UEMZ01000028.1	45,980…56,093	+	+	4B
*CHA1*_L_^T^-*MTL*α^T^-ZYRO0F18634g^T^	UEMZ01000013.1	263,261…275,557	+	–	–
**Yα^P^**					
*DIC1*^T^-*MTL*α^P^-*SLA2*^P^	UEMZ01000013.1	35,683…40,522	+	+	1D
*CHA1*_L_^T^-*MTL*α^P^-*SLA2*^P^	UEMZ01000003.1	11,848…18,890 (r.c)	+	+	2D
*CHA1*_L_^P^-*MTL*α^P^-ZYRO0F18634g^P^	UEMZ01000003.1	241,988…250,941 (r.c.)	+	+	5E
*DIC1*^T^-*MTL*α^P^-*SLA2*^N^	UEMZ01000007.1	1,444,839…1,449,671 (r.c.)	–	–	–
**Ya**					
*DIC1*^N^-*MTL*a^N^-*SLA2*^T^	UEMZ01000008.1	1,427,380…1,431,846	+	–	–
*CHA1*^T^-*MTL*a^T^-ZYRO0C18392g^T^	UEMZ01000015.1	1,296,432…1,304,606 (r.c.)	+	+	3C
*CHA1*^P^-*MTL*a^P^-ZYRO0C18392g^P^	n.r.	n.r.	+	+	6F

*MTL cassettes were found by BLAST searching Ya and Yα coding DNA sequences from Z. rouxii CBS732^*T*^ reference genome against DBG2OLC and MaSuRCA assemblies and then they were validated by long PCR and Sanger sequencing. JCM66020 MTL cassettes were described based on flanking genes according to nomenclature reported by Watanabe et al. (2017). Briefly, numbers 1–6 indicate 5′-flanking genes DIC1^*T*^, CHA1_*L*_^*T*^, CHA1^*T*^, DIC1^*P*^, CHA1_*L*_^*P*^, and CHA1^*P*^, respectively. Capital letters A–F indicate 3′-flanking genes SLA2^*T*^, ZYRO0F18634g^*T*^, ZYRO0C18392g^*T*^, SLA2^*P*^, ZYRO0F18634g^*P*^ and ZYRO0C18392g^*P*^, respectively. r.c, reverse complement; n.r., not reported.*

MaSuRCA assembly resulted in an assembled genome size of 21.09 Mb distributed across 59 scaffolds with *N*_50_ of 1.34 Mb (Table 3). In our previous analysis, 10,524 predicted genes were estimated by Exonerate (Slater and Birney, 2005; Bizzarri et al., 2018). Here, gene number was re-calculated for both DBG2OLC and MaSuRCA assemblies using YGAP software. Based on this analysis, DBG2OLC and MaSuRCA displayed roughly the same number of predicted genes (Table 3). Single-copy orthologs analysis by BUSCO 3.0 revealed a high degree of completeness in both assemblies (>98.0%), even if MaSuRCA retrieved more duplicated orthologs than DBG2OLC.

**Table 3:** Assembly metrics and annotation completeness obtained by using BUSCO universal fungal genes (saccharomycetales_odb9) data set.

Feature	Assembler	
	**DBG2OLC**	**MaSuRCA**
Assembly size (bp)	20,910,059	21,093,102
Number of scaffolds	33	59
G+C content (%)	39.65	39.95
*N*_50_ contig size (bp)	1,393,912	1,337,761
*N*_90_ contig size (bp)	400,395	638,558
Gaps	0	0
Longest scaffold (bp)	1,903,919	2,966,114
Number of genes	10,678	10,362
BUSCO complete genes	1,687 (98.6%)	1,692 (98.9%)
BUSCO duplicated genes	1,491 (87.1%)	1,582 (92.5%)

**FIGURE 1:**
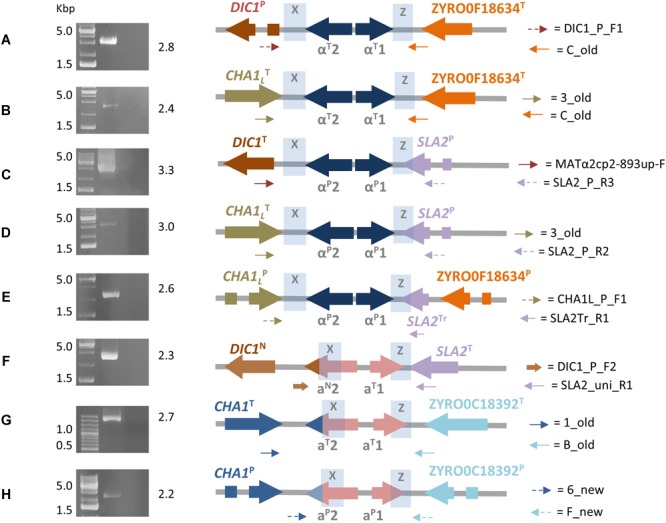
Final ATCC42981_R *MAT*-like cassettes organization resulting from DBG2OLC and MaSuRCA assemblies. Picture depicts full-length PCR results **(left)** and the corresponding inferred *MTL* arrangements **(right)**. Genes from T- and P-subgenomes are marked with T and P superscripts, respectively. *SLA2* truncated variant is marked with Tr superscript, while *DIC1* and *MAT*a2 new variants with N superscript. Blue shading denotes the X and Z regions. Dot arrows indicate P-variants of *MAT* flanking genes, while full arrows stand for T-variants. Variable *MAT*a2 3′-end tags are indicated as colored tips. Capital letters from A to H refer to primer pairs listed in Supplementary Table S1. Numbers on the right of gel electrophoresis images indicate PCR product length in Kbp.

MaSuRCA validated five out of eight DBG2OLC *MTL* cassettes, while one was MaSuRCA assembly specific (Table 2 and Supplementary Table S2). All six MaSuRCA cassettes were consistent with JCM22060. Like in DBG2OLC, MaSuRCA*-*derived *MTL* cassettes especially laid at the scaffold edges, confirming difficulties in scaffolding over repeated X and Z sequences shared by multiple and partially divergent *MTL*-flanking regions. Figure 1 showed that direct *in vitro* PCR validated eight *MTL* arrangements. Moreover, MaSuRCA consensus sequences were often more consistent with Sanger sequencing compared with DBG2OLC. Probably, this discrepancy resulted from a more aggressive DBG2OLC approach enabled to reduce the genome fragmentation, but at the price of local assembling accuracy.

#### *MTL*α^P^ Cassettes

Congruently with our previous data (Bizzarri et al., 2016), the DBG2OLC and MaSuRCA assemblies supported the cassettes *DIC1*^T^-*MTL*α^P^-*SLA2*^P^ and *CHA1*_L_^T^-*MTL*α^P^-*SLA2*^P^ (Table 2). PCR approach confirmed these arrangements (Figure 1). Pairwise comparisons showed that *DIC1*^T^ and *CHA1_L_*^T^ were 100% identical to the *Z. rouxii* CBS732^T^ counterparts. In cassette *DIC1*^T^-*MTL*α^P^-*SLA2*^P^, the 3′-flanking gene *SLA2*^P^ diverged from CBS732^T^ counterpart (83.65% identity), and resembled *SLA2* found in allodiploid NBRC110957 and NBRC1876 (99.58% identity) (Sato et al., 2017; Watanabe et al., 2017). In *CHA1*_L_^T^-*MTLα*^P^-*SLA2*^P^ cassette, DBG2OLC assembly reported mismatches compared to *SLA2*^P^ in NBRC110957 (93.12% identity), which were not supported by MaSuRCA. Sanger sequencing confirmed the accuracy of MaSuRCA assembling (Supplementary Figure S2).

According to the model of T- and P-subgenomes, *DIC1*^T^-*MTL*α^P^-*SLA2*^P^ and *CHA1*_L_^T^-*MTL*α^P^-*SLA2*^P^ should be chimeric cassettes arisen from rearrangements involving the X regions. NBRC110957 also contains the *DIC1*^T^-*MTL*a^P^-*SLA2*^P^ chimeric arrangement (Watanabe et al., 2017; Supplementary Table S2), suggesting that recombination is frequent upstream the Y sequence. Recombinant sites at the *MAT* locus were also documented in several *Saccharomyces* lager yeasts (Bond et al., 2004; Hewitt et al., 2014). Breakpoints frequently occurred at the right of the *MAT* locus resulting in hybrid *S. cerevisiae*–*S. eubayanus* chromosomes III. These chromosomes contain *S. eubayanus* sequences in the W region and *S. cerevisiae* in the Y region hitch-hiking downstream genes or *vice versa* (Monerawela and Bond, 2017). In lager yeast Ws34/70 a possible location for the recombination event is a 9-bp insertion in the *S. eubayanus* X region compared to *S. cerevisiae*. We found a similar indel between X regions of ATCC42981_R *DIC1* variants (Supplementary Figure S3), confirming that X region could represent a specific ‘fragile’ chromosomal location susceptible to double strand breakage (DSB).

Novel sets of P-subgenome-specific primers confirmed an additional *MTL*α^P^ locus (*CHA1_L_*^P^-*MTL*α^P^-ZYRO0F18634g^P^) which escaped our previous reconstruction (Bizzarri et al., 2016) (Figure 1). Based on Watanabe et al. (2017), this locus should be a cryptic *HML* cassette, which did not affect the true cell identity. This cassette had a truncated *SLA2* sequence downstream the Z region, confirming DNA erosion on the right side of *MAT* locus (Gordon et al., 2011). Interestingly, in both DBG2OLC and MaSuRCA assemblies this cassette is linked to *CHA1_L_*^T^-*MTL*α^P^-*SLA2*^P^ on the same scaffold (Supplementary Figure S4).

#### *MTL*α^T^ Cassettes

DBG2OLC and MaSuRCA assemblies failed to congruently reconstruct *MTL*α^T^ loci (Table 2). DBG2OLC scaffold UEMZ01000013.1 contains *CHA1*_L_^T^-*MTL*α^T^-ZYRO0F18634g^T^ linked to the chimeric cassette *DIC1*^T^-*MTL*α^P^-*SLA2*^P^, while another *MTL*α^T^ locus (*DIC1*^P^-*MTL*α^T^-ZYRO0F18634g^T^) lies on the scaffold UEMZ01000028.1. MaSuRCA assembly reported only the *DIC1*^P^-*MTL*α^T^-ZYRO0F18634g^T^ cassette. Moreover, *MTL* cassette linkage differed between DBG2OLC and MaSuRCA: *DIC1*^T^-*MTL*α^P^-*SLA2*^P^ was linked to *CHA1*_L_^T^-*MTL*α^T^-ZYRO0F18634g^T^ in DBG2OLC, while it was linked to *CHA1*_L_^T^-*MTLα*^P^-*SLA2*^P^ and *CHA1_L_*^P^-*MTL*α^P^-ZYRO0F18634g^P^ in MaSuRCA (Supplementary Figure S4). PCR approach supported both *MTL*α^T^ cassettes from DBG2OLC assembly (Figure 1), while scaffold comparison suggests that MaSuRCA collapsed the *CHA1*_L_^T^ flanking regions into a single locus (Supplementary Figure S4).

#### *MTL*a Cassettes

Blast search against the DBG2OLC assembly revealed two *MTL*a cassettes (Table 2). The arrangement *CHA1*^T^-*MTL*a^T^-ZYRO0C18392g^T^ was also supported by MaSuRCA and PCR approach, and was congruent with our previous reconstruction (Bizzarri et al., 2016) and with JCM22060 (Watanabe et al., 2017) (Supplementary Table S2).

**FIGURE 2:**
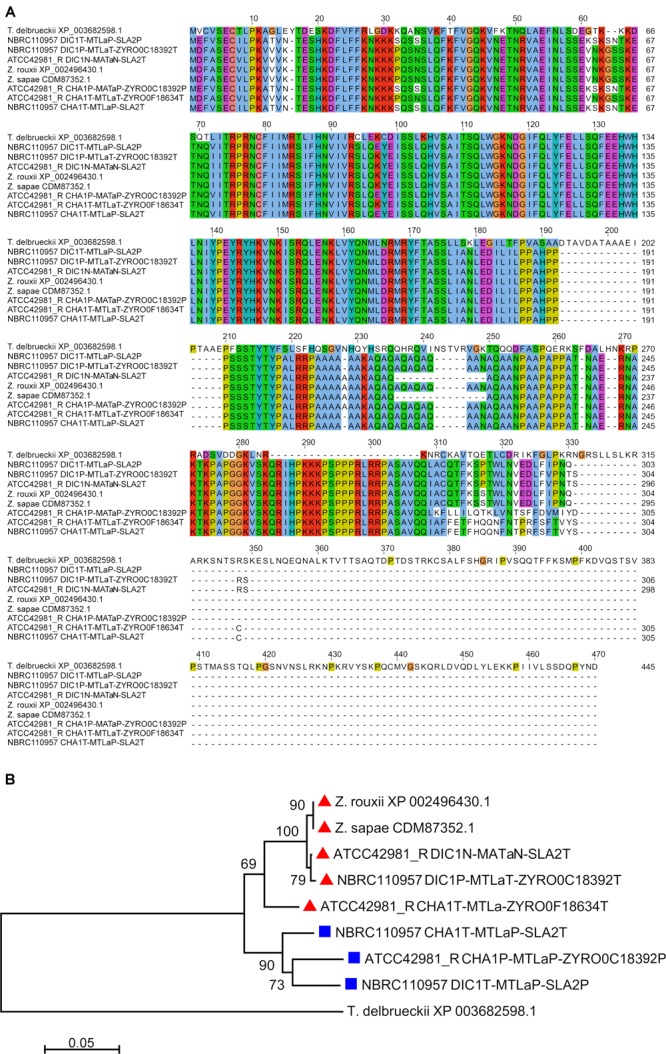
Multiple sequence alignment and phylogenetic analysis of MATa2 proteins. **(A)** Depicts the alignment involving 9 MATa2 amino acid sequences. The amino acid identities were colored according to Clustal Omega color scheme (Sievers and Higgins, 2014). In **(B)** dendrogram was inferred using the Neighbor-Joining method. The percentages of replicate trees in which the associated taxa clustered together in the bootstrap test (1,000 replicates) are shown next to the branches, when ≥50%. The evolutionary distances were computed using the p-distance method and are in the units of the number of amino acid differences per site. All positions containing gaps and missing data were eliminated. Red triangles and blue squares marked T and P variants.

The second *MTL*a locus resolved by DBG2OLC, *DIC1*^N^-*MTL*a^N^-*SLA2*^T^, contained a^T^1 and a novel a^N^2 gene variant (indicated with N superscript) which was 97.99% identical to *MAT*a2 from NBRC110957 *DIC*1^P^-*MTL*a^T^-ZYRO0C18392^T^ cassette (Figure 2). PCR approach demonstrated that this cassette really exists in ATCC42981_R genome, even if it was missing both in MaSuRCA assembly and in JCM22060 (Figure 1). Like in case of *SLA2*^P^ from *CHA1*_L_^T^-*MTL*α^P^-*SLA2*^P^, DBG2OLC *MAT*a2 sequence showed some indels in homopolymeric stretches compared to the Sanger-sequence data (98.54% pairwise identity), resulting in a prematurely interrupted ORF (data not shown). The neighbor genes at the 5′ and 3′ sides were a novel *DIC1* variant (named *DIC1*^N^) and the *SLA2*^T^ gene, respectively. Noteworthy, the *DIC1*-*MAT*-*SLA2* arrangement is retained around the transcriptionally active *MAT* loci in almost all the pre-WGD species (Gordon et al., 2011). Therefore *DIC1*^N^-*MTL*a^N^-*SLA2*^T^ cassette could be a good candidate to be the active *MAT*a cassette in ATCC42981_R.

Finally, PCR approach with haplotype P-specific primers identified a third *MTL*a locus (*CHA1*^P^-*MTL*a^P^-ZYRO0C18392g^P^) which was present in JCM22060 and in MaSuRCA assembly (Table 2). Blast search for *CHA1*^P^ gene revealed that DBG2OLC assembler did not extend scaffold UEMZ01000005.1 beyond this gene.

### Reconstruction of *MTL* Structure

**FIGURE 3:**
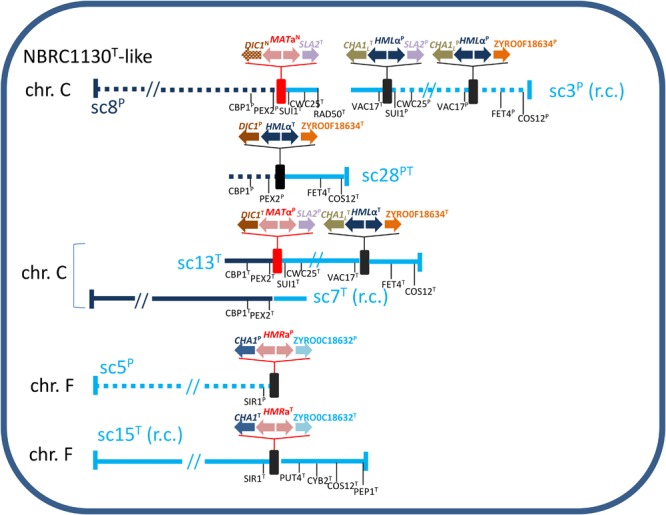
Inferred gene organization around the *MTL* loci in ATCC42981_R. Scaffold (sc) numbers referred to the DBG2OLC genome assembly deposited in European Nucleotide Archive under accession number PRJEB26771 (Bizzarri et al., 2018); for brevity each scaffold is identified by the last number of ENA code (*i.e.*, UEMZ01000028.1 in short sc28). Solid and dotted lines referred to T- and P-subgenomes, respectively. Genes from T- and P-subgenomes are marked with T and P superscripts, respectively, while *DIC1* and *MAT*a2 new variants with N superscript. Red and black rectangles defined *MAT* and *HML*/*HMR* loci, respectively. Scaffold lengths are not in scale. r.c., reverse complement.

Analysis of regions around *MTL* loci assisted us to reconstruct the putative *MTL* structure in ATCC42981_R. NBRC1130^T^ culture retains ancestral *MTL* arrangement compared with CBS732^T^ (Watanabe et al., 2013) and was used as reference strain. In this strain, chromosome C contains *MAT* and *HML* loci flanked by sets of genes which were also conserved around ATCC42981_R *MTL* cassettes (Supplementary Figure S5). In particular, *MAT* locus was flanked on the left by *PEX2* and *CBP1* and on the right by *SUI1* and *CWC25*, while *HML* cassette was flanked by *VAC17* at the left side and by *FET4* and *COS12* at the right side (Figure 3). Blast analysis indicated that DBG2OLC scaffold UEMZ01000008.1 was almost collinear to NBRC1130^T^ chromosome C in the first 1,427,380 bp. Genes upstream and downstream the *MAT*a^N^ cassette were P and T-type, respectively. Congruently, *MAT*a^N^ cassette retained the synteny with *PEX2*^P^ and *CBP1*^P^ at 5′- and *SUI1*^T^ and *CWC25*^T^ at 3′-end. However, 3′-end side was interrupted at *RAD50*^T^. Scaffold UEMZ01000003.1 (rc) linked *CHA1*_L_^T^-*MTL*α^P^-*SLA2*^P^ and *CHA1_L_*^P^-*MTL*α^P^-ZYRO0F18634g^P^ cassettes (Figure 3). Reciprocal translocation between chromosomes C from T and P haplotypes led to a similar arrangement in CBS4837 (Watanabe et al., 2017). As result, in CBS4837 the *MAT*α^P^ expression cassette is linked to *CHA1*_L_^T^-*MTLα*^P^-*SLA2*^P^ and *CHA1_L_*^P^-*MTL*α^P^-ZYRO0F18634g^P^. In ATCC42981_R, flanking gene analysis also supported a linkage between *MAT*a^N^ and *CHA1*_L_^T^-*MTLα*^P^-*SLA2*^P^/*CHA1_L_*^P^-*MTL*α^P^-ZYRO0F18634g^P^ cassettes, suggesting that scaffolds UEMZ01000008.1 and UEMZ01000013.1 contributed to the chimeric chromosome C. Like in CBS4837 (Watanabe et al., 2017), this chromosome C could arise from a reciprocal translocation between two ancestral T and P chromosomes C.

Scaffold UEMZ01000028.1 was chimeric with P-type (*PEX2* and *CBP1*) and T-type (*FET4* and *COS12*) genes upstream and downstream the cassette *DIC1*^P^-*MTL*α^T^-ZYRO0F18634g^T^, respectively (Figure 3). The loss of gene block between *MAT* and *HML* cassettes suggested that a deletion between *MAT* and *HML* cassettes led to this arrangement, similar to that described in strain NBRC0686 (Watanabe et al., 2013; Supplementary Figure S5). Alternatively, in CBS4837 a similar arrangement resulted from reciprocal translocation leading to chimeric chromosome C (Watanabe et al., 2017).

DBG2OLC scaffold UEMZ01000013.1 exhibited T-type flanking genes around *DIC1*^T^-*MTL*α^P^-*SLA2*^P^ and *CHA1_L_*^T^-*MTL*α^T^-ZYRO0F18634g^T^. Overlapping region with scaffold UEMZ01000007.1 suggested that scaffolds UEMZ01000013.1 and UEMZ01000007.1 could contribute to the T-type chromosome C in ATCC42981_R (Figure 3).

NBRC1130^T^ strain has the *HMR*a locus on chromosome F. *SIR1* and a set of genes including *PUT4, CYB2, COS12*, and *PEP1* are upstream and downstream to *HMR*a, respectively (Supplementary Figure S5). ATCC42981_R DBG2OLC assembly exhibited two scaffolds retaining this synteny, namely 5 and 15 (rc). Scaffold UEMZ01000005.1 contained P-type genes, including *SIR1*^P^ (Figure 3). Unfortunately, DBG2OLC assembler interrupted this scaffold after *CHA1*^P^. However, MaSuRCA assembly retained *PUT4*^P^, *CYB2*^P^, *COS12*^P^, and *PEP1*^P^ downstream of *HMR*a^P^, suggesting that ATCC42981_R has a P-type chromosome F collinear to NBRC1130 chromosome F. Syntenic relationships and Blast analysis supported scaffold UEMZ01000015.1 as the T-type version of NBRC1130^T^ chromosome F (Supplementary Figure S5).

### Disclosing the True Cell Identity

**FIGURE 4:**
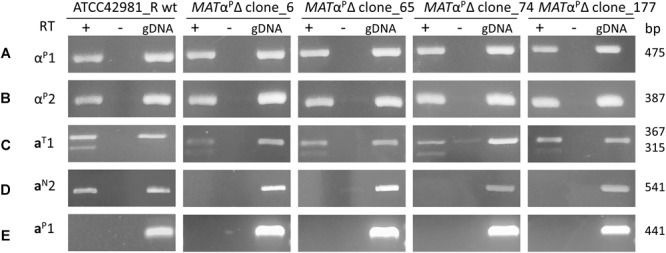
Gene expression at the *MAT* loci in ATCC42981_R wild type and *MAT*α^P^Δ deletion mutants. cDNA was amplified from total RNA extracted from stationary growing cells. Plus or minus indicates with or without reverse transcriptase in cDNA synthesis reaction, respectively. Gene variants from T- and P-subgenomes are indicated with T and P superscripts, while divergent *MAT*a2 gene variant from *DIC1*^N^-*MAT*a^N^-*SLA2*^T^ cassette with N superscript. Capital letters from A to E refer to primer sets listed in Supplementary Table S1. wt, wild type; gDNA, genomic DNA.

Watanabe et al. (2017) identified two *MTL* patterns: strains with pattern A, such as NBRC110957, exhibit two active *MAT* loci, namely *DIC1*^T^-*MAT*^P^-*SLA2*^P^ and *CHA1*^T^-*MTL*^P^-*SLA2*^T^, while strains with pattern B have *DIC1*^T^-*MAT*^P^-*SLA*2^P^ as active *MAT* locus, even if they also actively transcribed genes from *CHA1_L_*^T^-*MTL*^P^-*SLA*2^P^. JCM66020 belongs to this last group, exhibits a *MAT*α^P^ idiomorph and, congruently, mates only the tester strain a (CBS4838). Conversely, ATCC42981_R displays another pattern of putatively active *MAT* loci, namely, *DIC1*^T^-*MAT*α^P^-*SLA*2^P^ and *DIC1*^N^-*MAT*a^N^-*SLA2*^T^, in addition to the *CHA1_L_*^T^-*MTL*^P^-*SLA*2^P^ cassette. RT-PCR analysis confirmed that α^P^1, α^P^2, a^N^2 and a^T^1 genes were expressed, while a^P^1 gene encoded by *CHA1*^P^-*MTL*^P^-ZYRO0C18392g^P^ cassette was silent (Figure 4). Interestingly, a^T^1-specific RT-PCR resulted in two PCR amplicons compatible with alternative spliced intronic sequence.

Genome comparison with other pre-WGD yeasts indicates that *HML*α silent cassettes are generally 5′-flanked by *CHA1_L_* (Gordon et al., 2011). Conversely, strains with pattern B actively transcribed *MTL* genes from *CHA1_L_*^T^-*MTL*-*SLA2*^P^ cassette without that these transcripts affect cell identity (Watanabe et al., 2017). This is evident for strain CBS4837, where genes encoding opposite α^P^ and a^P^ idiomorphs are both expressed by *DIC1*^T^-*MAT*^P^-*SLA*2^P^ and *CHA1_L_*^T^-*MTL*^P^-*SLA2*^P^ cassettes, respectively. In JCM22060 (encoding α^P^ genes at both these loci), outcross experiment with CBS4837 and gamete segregation support that cell identity was determined by *DIC1*^T^-*MAT*^P^-*SLA*2^P^ cassette. To establish which cassette contributes to cell identity in ATCC42981_R, we deleted α^P^ idiomorph genes by replacing the entire segment including α^P^1, α^P^2 encoding genes and the intergenic region from *DIC1*^T^-*MAT*α^P^-*SLA*2^P^ with *loxP*-*kanMX*-*loxP* module. From approximatively 300 screened colonies we obtained four G418^R^ clones. PCR genotyping showed that these clones are *MAT*α^P^Δ deletants containing *loxP*-*kanMX*-*loxP* surrounded by *DIC1*^T^ and *SLA*2^P^ instead of *MAT*α^P^ locus (Supplementary Figure S1).

Gene deletion of *DIC1*^T^-*MAT*α^P^-*SLA*2^P^ cassette should abolish the heterozygosity at the *MAT*a/α active loci and results in an allodiploid partially resembling a haploid cell with a mating-type. Conversely, ATCC42981_R *MAT*α^P^Δ still showed α^P^1 and α^P^2 gene expression (Figure 4). These mRNAs could be only transcribed by the not completely silenced cassettes *CHA1_L_*^T^-*MTL*α^P^-*SLA2*^P^ or by *CHA1_L_*^P^-*MTL*α^P^-ZYRO0F18634g^P^.

Since allodiploid lacking one *MAT* active locus should behave like haploid with opposite mating-type, we expected to detect both a1 and a2 transcripts in ATCC42981_R *MAT*α^P^Δ mutants. In some haploid pre-WGD species, a2 gene encodes a transcription activator of asgs, while a1 should not affect asgs in a cells (Tsong et al., 2003, 2006; Baker et al., 2012). Unexpectedly, RT-PCR showed that *MAT*α^P^ deletion switched off a2 but not a1 gene expression (Figure 4). By contrast, ATCC42981_R wild type both transcribed a1 and a2 genes. Preliminary end-point RT-PCRs showed that the asgs *AGA2, STE6*, and *STE2* are transcriptionally active in both wild type and *MAT*α^P^Δ cells (data not shown).

### Mating and Sporulation Competence Assays

**FIGURE 5:**
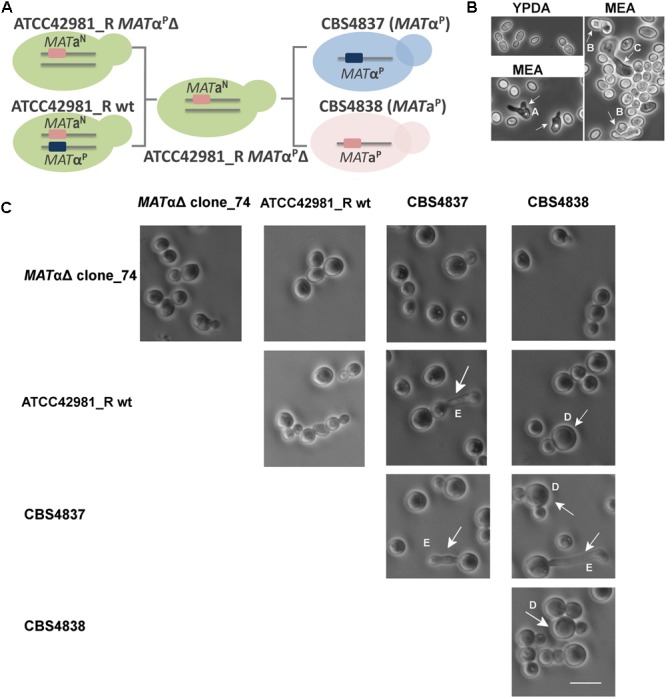
Self- and out-cross fertility assays of ATCC42981_R wild type and *MAT*α^P^Δ deletion mutant. **(A)** Shows the scheme of self- and out-cross fertility assays involving ATCC42981_R wild type and *MAT*αΔ clone_74, while **(B)** depicts representative phase contrast microscopic images of control strain *Zygosaccharomyces parabailii* G21C grown in YPDA and MEA media. **(C)** Shows selected phase contrast microscopic images of representative ATCC42981_R *MAT*αΔ clone_74 grown as monoculture and in mixture with ATCC42981_R wild type, CBS4837 (α) and CBS4838 (a). Phase contrast microscopic images of ATCC42981_R wild type, CBS4837 and CBS4838 monocultures were reported for comparative purposes. The scale bar represents 11 μm and was reported in only one picture for brevity. Capital letters from A to E indicate abortive shmoo, conjugated asci with ascospores, conjugative bridge, giant cells, and abnormal conjugative tube, respectively. All cells were photographed after 7 days on incubation on MEA medium at 27°C. wt, wild type.

To test whether the *MAT*α^P^ deletion rescues the mating competence in ATCC42981_R, we carried out self- and out-cross fertility assays of the wild type strain and the *MAT*α^P^Δ transformants as monoculture or in mixture with CBS4837 (α) or CBS4838 (a) mating testers, respectively. If *MAT*α^P^Δ transformants behave as homothallic haploids, they should produce shmoo and conjugated asci as monoculture, while, if they are like heterothallic haploids, they should mate and sporulate in mixture either with CBS4837 or CBS4838. We used three media reported in literature to promote zygote formation and conjugated asci of *Zygosaccharomyces* cells, as proved for *Z. parabailii* G21C (Figure 5). In particular, 5-6% NaCl addition was reported to increase sporulation occurrence (Mori and Onishi, 1967). Like the wild type strain, *MAT*α^P^Δ mutants did not show any evidence of conjugative bridge and/or conjugative asci either as monoculture or in mixture with the mating testers (Figure 5). The composition of three test media did not affect the inability to mate or to undergo meiosis. Overall, these evidences indicate that the deletion of active *MAT*α^P^ locus did not make ATCC42981_R cells phenotypically heterothallic or homothallic haploids.

## Discussion

Our study is the first to combine the Nanopore whole-genome sequencing to conventional PCR-based methods in order to survey *MTL* loci in a *Z. rouxii* allodiploid genome. This yeast is particularly prone to outbreeding and provides a particularly appealing platform to study genome re-shaping after the merger of two parental subgenomes. Recombination and introgression between subgenomes have been rampant in hybrid yeasts, resulting in loss of heterozygosity and gradual genome reduction (Sipiczki, 2008). In *Z. rouxii MTL* loci markedly contribute to this genomic plasticity (Watanabe et al., 2013; Solieri et al., 2014). As consequence, this species frequently undergoes chromosomal translocations at the *MTL* loci, which make hard the understanding of true cell identity by simple *MTL* genotyping. For example, haploid *Z. rouxii* strain CBS732^T^ switched mating-type at the *CHA1*-*MAT*-*SLA2* locus (Bizzarri et al., 2018), suggesting that *CHA1* gene flanks the actively transcribed *MAT* locus instead of *DIC1*. Several assortments of different flanking gene variants and distinct idiomorph encoding genes make challenging and laborious to resolve the complex genetic *MTL* architecture by PCR targeted approaches. For these reasons, we generated a high-quality genome assembly in order to dissect complex rearrangements at the *MTL* loci that were not fully resolvable from the earlier survey based only on long-range PCR amplification (Bizzarri et al., 2016). One of the major advantages of the ONT is the possibility of sequencing very long DNA fragments, which span the entire *MTL* cassettes. This strategy assures to accurately reconstruct gene order around different *MTL*s. On the other hand, using noisy ultra-long reads for self-correction and assembling of highly heterozygous genomes can affect the consensus sequence accuracy and the parental haplotypes sorting. In case of ATCC42981_R, distinguishing between homeologous sequences is further challenging as only the *Z. rouxii* parental genome is available to guide homeologous scaffold assembly. Error rate made necessary to polish MinION reads with Illumina-derived reads, resulting into DBG2OLC-driven hybrid *de novo* genome assembly (Bizzarri et al., 2018). However, our result showed that a single “best assembler” does not exist to resolve highly heterozygous and highly repeated *MTL* regions. DBG2OLC assembly suffers from poor performance in certain sequence contexts, such as in regions with low coverage or regions that contain short repeats. Besides, the new assembly generated with MaSuRCA showed higher sequencing accuracy compared to DBG2OLC, but loses some *MTL* cassettes. As bottom-end validation step, PCR approach was used to discard artificial *MTL* arrangements arisen from flawed contig assemblies. This strategy resolves controversies over *MTL* loci in ATCC42981_R genome derived from the analysis of the Japanese stock JCM22060 (Watanabe et al., 2017).

Reconstruction of *MTL* structure indicates that ATCC42981_R resembles CBS4837 for the exception of an additional scaffold containing *DIC1*^T^-*MTL*α^P^-*SLA2*^P^ linked to *CHA1*_L_^T^-*MTL*α^T^-ZYRO0F18634g^T^ (Figure 3). This assessment was congruent with previous PFGE-Southern blotting which showed two signals for *MAT*α-specific probe (Bizzarri et al., 2016). The most significant difference between ATCC42981_R and JCM22060 is that ATCC42981_R harbors the transcriptionally active *MAT*a^N^ cassette in addition to the expected *MAT*α^P^. Differently from *Z. parabailii* (Ortiz-Merino et al., 2017), *MAT*a^N^ cassette of ATCC42981_R contains *MAT*a1 gene. This means that *Z. rouxii* retains the ancestral regulatory circuit based on a1–α2 heterodimer as diploid cell sensor (Booth et al., 2010). Watanabe et al. (2017) showed that strain JCM22060, which contains only *MAT*α^P^, mates the tester strain a in a medium containing Shoyu-koji extract. By contrast, we did not find any evidence of meiosis or mating in ATCC42981_R (Bizzarri et al., 2016), when grown on the media reported in literature to promote *Z. rouxii* mating and sporulation (James and Stratford, 2011). Watanabe et al. (2017) argued that difference in medium composition could account for the phenotypic discrepancy between ATCC42981_R and the sister stock JCM22060. As the Shoyu-koji extract is difficult to gain in western countries, we cannot rule out this hypothesis. Otherwise, heterozygosity at the *MAT* locus could significantly contribute to the allodiploid infertility. In particular, the hybrid heterodimer with divergent a1 and α2 subunits brings the cell in an ‘haploid-diploid intermediate’ functional state which hamper both the meiosis commitment and the responsiveness to mating stimuli (Bizzarri et al., 2016).

In *Saccharomyces* clade, experimental deletion of one *MAT* locus leads to allotetraploids suitable to undergo meiosis (Greig et al., 2002; Pfliegler et al., 2012). Similarly, *Z. parabailii* and *Z. pseudobailii* hybrid strains ATCC60483 and MT15 were recently supposed to be fertile due to the accidental breakage of 1 of the 2 homeologous copies of the *MAT* locus (Ortiz-Merino et al., 2017; Braun-Galleani et al., 2018). A prediction of this model is that artificial deletion of one *MAT* locus in *Zygosaccharomyces* cells should override the arrest in mating commitment. In our model, ATCC42981_R cells did not behave as haploids with idiomorph a, when the *MAT*α^P^ locus was deleted. This suggests that mechanism underpinning the cell identity in *Z. rouxii* hybrids could be different from those involved in cell identity regulation of the sister species *Z. parabailii* and *Z. pseudobailii*.

Gene deletion of transcriptionally active *MAT*α^P^ locus did not rescue the ability to produce conjugated asci in ATCC42981_R, while the persistence of α1 and α2 transcripts suggests that *HML*α silencing was leaky in ATCC42981_R. Consequently, α^P^ genes either from *CHA1*_L_^T^-*MTL*α^P^-*SLA2*^P^ or *CHA1_L_*^P^-*MTL*α^P^-ZYRO0F18634g^P^ are transcriptionally active in *MAT*α^P^Δ mutants. Strain NBRC110957, which does not have the *CHA1*_L_^T^-*MTL*α^P^-*SLA2*^P^ cassette, uses *CHA1_L_*^P^-*MTL*α^P^-ZYRO0F18634g^P^ as donor during switching from a^P^ to α^P^ (Watanabe et al., 2017). This suggests that *CHA1_L_*^P^-*MTL*α^P^-ZYRO0F18634g^P^ cassette is most likely silenced and that α^P^ could be expressed by the *CHA1*_L_^T^-*MTL*α^P^-*SLA2*^P^ in ATCC42981_R. Congruently, strain CBS4837 actively transcribed genes from *CHA1*_L_^T^-*MTL*a^P^-*SLA2*^P^ cassette. These findings make less probable the alternative hypothesis that *MAT*α^P^ deletion induces *HML*α cassette de-silencing. Abnormal expression of cryptic *HMR*/*HML* loci has been described in *Vanderwaltozyma polyspora*, the *Z. rouxii* closest relative that branched after WGD (Roberts and Van der Walt, 1959). Consequently, *V. polyspora* haploid cells behave as a/α diploid and appear mating-incompetent for many generations only to subsequently restore silencing. Significantly, *V. polyspora* lacks of Sir1 histone deacetilase, which mediates the *HM* loci silencing in *S. cerevisiae* together with the SIR complex (Sir2/Sir3/Sir4). In *S. cerevisiae* failure to recruit Sir1 is thought to account for the instability of subtelomeric silencing relative to *HM* loci (Chien et al., 1993). Like *V. polyspora, Candida glabrata* is another species close to *Z. rouxii*, which lacks of a *SIR1* ortholog (Gabaldón et al., 2013). A defective silencing system leads to the expression of *MAT*a genes in *C. glabrata MAT*α cells (Muller et al., 2008) and makes *HML* more prone to HO cleavage at the Y/Z junctions (Boisnard et al., 2015). *Z. rouxii* has the archetypal member of the *SIR1* family, *KOS3* (*K*in *o*f *S*ir1 3) (Gallagher et al., 2009). In pre-WGD species *Torulaspora delbrueckii KOS3* located ∼1 kb away from *HMR* and plays a key role in *HML*/*HMR* silencing (Ellahi and Rine, 2016). Strikingly, in ATCC42981_R we also found two *KOS3* copies, *KOS3*^T^ and *KOS3*^P^, upstream of *HMR*a^T^ and *HMR*a^P^ loci, respectively. In addition, Sir1 and the components of SIR complex have been reported to rapidly evolve in the Saccharomycetaceae family. This could potentially jeopardize the efficiency of the silencing machinery in interspecific hybrids. For example, Sir1, Sir4 and the *cis*-acting silencer sequences are incompatible in *S. cerevisiae* × *S. uvarum* hybrids (Zill et al., 2010, 2012). In ATCC42981_R, heterochromatin formation across silent loci could be less effective due to the incompatibility in the silencing machinery between the T- and P-subgenomes. Watanabe et al. (2017) suggest that chimeric *MTL* cassettes could display epigenetic expression control when only E silencer sequence is maintained around *MTL* locus. This could produce allodiploid single cells which undergo epigenetic silencing at one of *MAT* loci and restore fertility. In ATCC42981_R two *DIC1*-*MAT*-*SLA2* cassettes assure active transcription of opposite idiomorphs, while the presence of E silencer only at the right side of *HML*α^P^ locus could unlock the silencing and mask the loss of heterozygosity at the *MAT* locus induced by *MAT*α locus deletion.

Strikingly, the depletion of α^P^1 and α^P^2 genes switched off the a2 but not the a1 gene transcription. Moreover in both deleted and wild type strains two a1 alternative spliced isoforms are present, one of them compatible with the retention of first intron. In *S. cerevisiae* exon–intron structure is conserved and the retention of first intron resulted in a functional a1 transcriptional factor that prevents mating (Ner and Smith, 1989). Since α1 activates the αsgs in the ancestral circuit of yeast cell identity (Baker et al., 2011), we rule out the possibility that α1 is involved in a2 gene repression. In *S. cerevisiae*, α2 represses asgs by binding asgs *cis*-regulatory sequences cooperatively with a MADS-box transcription regulator, Mcm1 (Tsong et al., 2003). *Z. rouxii*, which branched from the *S. cerevisiae* lineage prior to the loss of a2 gene, should maintain both the a2 activation and the α2 repression of asgs (Tsong et al., 2006; Baker et al., 2012). In *Lachancea kluyveri* haploid cells, α2 deletion induces the transcription of the asgs *AGA1* and *AGA2*, while a2 deletion decreases the asgs transcript levels (Baker et al., 2012). However, to the best of our knowledge, no evidence has been provided until now about the consequences of α2 gene deletion in diploid cells which retain a2 gene. As a1 is still expressed in *Z. rouxii MAT*αΔ/*MAT*a hemizygous cells, we speculate that a2 silencing could be a promoter-driven event directly or indirectly regulated by α2. Furthermore, in our *MAT*αΔ/*MAT*a model, the asgs were expressed even when a2 was switched off by the *MAT*α2 deletion, suggesting the existence of a different asgs regulatory network in the ATCC42981_R hybrid compared to *Z. rouxii*.

## Conclusion

This study revised the pattern of *MTL* loci in allodiploid strain ATCC42981_R. By taking advantage from ONT technology, we captured a novel *MAT*a cassette which did not correspond to the expected T and P counterparts, providing preliminary evidences that a third haplotype contributes to this genome. The differences between ATCC42981_R and JCM22060 support that *MTL*s are a root source of genetic variation, leading to novel chimeric *MTL* cassettes, different cell identities and, consequently, distinct phenotypic behaviors. While further researches are required to investigate mechanisms responsible of this extensive *MTL* reshaping, our results confirm that these yeast stocks are genetically unstable (Watanabe et al., 2013; Bizzarri et al., 2018). We also demonstrated how *HMR*/*HML* silencing is crucial to establish the cell identity, as leakage in *HML* silencing prevents allodiploid *MAT*α^P^Δ cells to behave like haploids. How allodiploid cell modulates a2 expression via α2 transcriptional factor represents an unexplored regulatory circuit that has to be investigated in future.

## Data Availability

The whole genome sequence datasets generated for this study can be found under the NCBI BioProject number PRJEB26771.

## Author Contributions

SC and LS contributed conception and design of the study. MB conducted the experiments described in this study. LB contributed to *in vitro* PCR validation and asg expression. HS and MD contributed to deletion mutant construction. SC and LP performed bioinformatic analysis of the whole genome sequence data. LS wrote the manuscript. SC and MB contributed to draft revision. All authors read and approved the final manuscript.

## Conflict of Interest Statement

The authors declare that the research was conducted in the absence of any commercial or financial relationships that could be construed as a potential conflict of interest.

## Footnotes

1 http://wolfe.ucd.ie/annotation/
