# Supplementary material for: Interplay of Chimeric Mating-Type Loci Impairs Fertility Rescue and Accounts for Intra-Strain Variability in Zygosaccharomyces rouxii Interspecies Hybrid ATCC42981
Source: Front Genet. 2019 Mar 1;10:137. doi: 10.3389/fgene.2019.00137 (PMC6405483; doi:10.3389/fgene.2019.00137)
Supplement: Supplementary file 1 [file Table_1.docx]

Supplementary Material

Interplay of chimeric mating-type loci impairs fertility rescue and accounts for intra-strain variability in *Zygosaccharomyces rouxii* interspecies hybrid ATCC42981

Melissa Bizzarri^1^, Stefano Cassanelli^1*^, Laura Bartolini^1^, Leszek P. Pryszcz^2^, Michala Dušková^3^, Hana Sychrová^3^, Lisa Solieri^1*^

*** Correspondence:** Lisa Solieri: lisa.solieri@unimore.it

# Supplementary Tables

**Table S1. Primer sets used in the present study.** Primers named as old are from Watanabe et al. (2013), whereas primers named as new derive from Watanabe et al. (2017). DNA sequences complementary to the *kanMX* gene sequence and plasmid sequences are written in lower-case.

| **Primer name** | **Sequence (5’ to 3’)** | **Corresponding Figure** |
| --- | --- | --- |
| DIC1_P_F1 | GGTATTTGATGGGAGCAGCA | Figure 1A |
| 3_old | TACTTACTGGATGAATCTTCTGTGA | Figure 1B |
| C_old | TCAGTACCAGAAGTGGTCTTTGAAA | Figures 1A, 1B |
| MATα2cp2-893up-F | CGGTAACGACTGTATAGCTAAG | Figures 1C, S1 |
| SLA2_P_R3 | GGACAGTTGGGAGACACTGAA | Figure 1C |
| 3_old | TACTTACTGGATGAATCTTCTGTGA | Figure 1D |
| SLA2_P_R2 | GGGAGACACTGAAGCGTTAGAT | Figure 1D |
| CHA1L_P_F1 | AGACAGCTACAAGGTGTTGTGA | Figure 1E |
| SLA2Tr_R1 | AAAGTCCTATTCACGTGACGAA | Figure 1E |
| DIC1_P_F2 | GAGTGGTATGGTGAAGCTGTG | Figure 1F |
| SLA2_uni_R1 | ATATATCTTATCGAGACAGTGTATTTC | Figure 1F |
| 1_old | GCTACTCCCTCATTAGAACATGAAA | Figure 1G |
| B_old | TCTATTTCGTCCGTTTATCGTTGGT | Figure 1G |
| 6_new | TGTATTGACCAGCTTCGTTTGA | Figure 1H |
| F_new | ATGGACTACACGTACCACAA | Figure 1H |
| 301_MATa1F7 | TGGATCTTAGACAGTGGTGTAAGG | Figure 3A |
| 301MATα1α2cp2R2 | TGTCATCCACATTGAAATCATCTC | Figure 3A |
| 301MATa1a2-cp2F1 | GTTTAGATGCCAGTGCTCTTCA | Figure 3B |
| 301MATa1a2-cp2R1 | GACACATTGCATTCTGTTAAACGT | Figure 3B |
| 301_MATA1F3 | GTAGCTTCCACAAGGTCTTCAAG | Figure 3C |
| 301_MATA1R4 | GCTGCTACAGCTTCCCTTTC | Figure 3C |
| 301_MATA2F8 | AGCCAAGTGGGCGATTTA | Figure 3D |
| 301_MATA2R2 | CATGTGTCTGCAATCACTTCAC | Figure 3D |
| MATA1_008_F1 | ATTCTCCAAATGATCTTCAGA | Figure 3E |
| MATA1_008_R1 | ATACCCATATTCTTACTTGAAGT | Figure 3E |
| MATα1/2cp2-kanMX-F-80nt | CATGTTTGAACGAGTGTTTTGTTCATTGGTTTGGAATAAACAGGTCTTCGACGTTTAGCCATGTCGAGGATTTAAACGTTTGACAttcgtacgctgcaggtcgac | Figure S1 |
| MATα1/2cp2-kanMX-R-80nt | CAACCGGTAAGTGTTCTTTCAATAAGTCAGTTGTGCAATGAAGTGGCAAGTCAGTTTTTAAGCAACACACCGCACGTACCGgcataggccactagtggatctg | Figure S1 |
| kanMX-R1 | CTCTGGCGCATCGGGC | Figure S1 |
| kanMX-F1 | CATTTGATGCTCGATGA | Figure S1 |
| MATα1cp2-374down-R | CCAAACTTTATGGATATGAGTTCTAGC | Figure S1 |
| AGA2_backbone1_rouxii_F | CATGTACCACTGTACCCAGTAAG | Data not shown |
| AGA2_backbone1_rouxii_R | ACCGTAGTAGTCCCGATTGA |  |
| STE2_backbone15_rouxii_F | CCTATTGGCCTCGTCTGTTAAT |  |
| STE2_backbone15_rouxii_R | TAGGCGGACAAGATATGAGGT |  |
| STE2_backbone5_no_rouxii_F | CCTATTGGCCTCGTCTGTTAAT |  |
| STE2_backbone5_no_rouxii_R | TAGGCGGACAAGATATGAGGT |  |
| STE6_backbone1_rouxii_F | TAACACTACCAGTGGGTAA |  |
| STE6_backbone1_rouxii_R | TCATAAGTGGACGTTTTGAAA |  |
| STE6_backbone6_no_rouxii_F | ATATCAAAATCGATGGCATGGA |  |
| STE6_backbone6_no_rouxii_R | AGCGGTTATTTTGTTGCCT |  |

**Table S2**. **Inventory of *MTL* cassettes in ATCC42981_R draft genome derived from DBG2OLC and MaSuRCA *de novo* assemblies.** DBG2OLC scaffolds (accession numbers: UEMZ01000001.1-UEMZ01000033.1) were derived from ATCC42981_R BioProject PRJEB26771. Grey shadow indicates *MTL* cassettes found in both the assemblies. JCM66020 *MTL* cassettes were described according to the nomenclature reported by Watanabe et al. (2017). Briefly, numbers from 1 to 6 indicate 5’ *MTL*-flanking genes *DIC1*^T^, *CHA1*_L_^T^, *CHA1*^T^, *DIC1*^P^, *CHA1*_L_^P^, and *CHA1*^P^, respectively. Capital letters A to F indicate 3’ *MTL*-flanking genes *SLA2*^T^, ZYRO0F18634g^T^, ZYRO0C18392g^T^, *SLA2*^P^, ZYRO0F18634g^P^ and ZYRO0C18392g^P^, respectively. Abbreviation: r.c., reverse complement.

| **Cassettes** | **Bizzarri *et al*. (2016)** | **JCM22060** | **PCR** | ***In silico* analysis** | **NBRC110957 (Accession number)** |
| --- | --- | --- | --- | --- | --- |
| **Yα^T^** |  |  |  |  |  |
| *DIC1*^P^-*MTL*α^T^-ZYRO0F18634g^T^ | - | 4B | + | DBG2OLC, MaSuRCA | *CHA1*^P^-*MTL*α^P^-ZYRO0C18392g^P^ (BDGX01000045) |
| *CHA1*_L_^T^-*MTL*α^T^-ZYRO0F18634g^T^ | + | - | + | DBG2OLC | *CHA1*_L_^T^-*MTL*α^T^-ZYRO0F18634g^T^ (BDGX01000025) |
| **Yα^P^** |  |  |  |  |  |
| *DIC1*^T^-*MTL*α^P^-*SLA2*^P^ | + | 1D | + | DBG2OLC,MaSuRCA | *DIC1*^T^-*MTL***a**^P^-*SLA2*^P^ (BDGX01000009) |
| *CHA1*_L_^T^-*MTL*α^P^-*SLA2*^P^ | + | 2D | + | DBG2OLC,MaSuRCA | - |
| *CHA1*_L_^P^-*MTL*α^P^-ZYRO0F18634g^P^ | - | 5E | + | DBG2OLC,MaSuRCA | *CHA1*_L_^P^-*MTL*α^P^-ZYRO0F18634g^P^ (BDGX01000013) |
| *DIC1*^T^-*MTL*α^P^-*SLA2***^N^** | - | - | - | DG2OLC | *-* |
| **Ya** |  |  |  |  |  |
| *DIC1***^N^**-*MTL***a^N^**-*SLA2*^T^ | + (partially) | - | + | DBG2OLC | *DIC1*^P^-*MTL***a**^T^-ZYRO0C18392g^T^ (BDGX01000035) |
| *CHA1*^T^-*MTL***a**^T^-ZYRO0C18392g^T^ | + | 3C | + | DBG2OLC,MaSuRCA | *CHA1*^T^-*MAT***a**^P^-*SLA2*^P^ (BDGX01000021) |
| *CHA1*^P^-*MTL***a**^P^-ZYRO0C18392g^P^ | - | 6F | + | MaSuRCA | - |

# Supplementary Figures

**
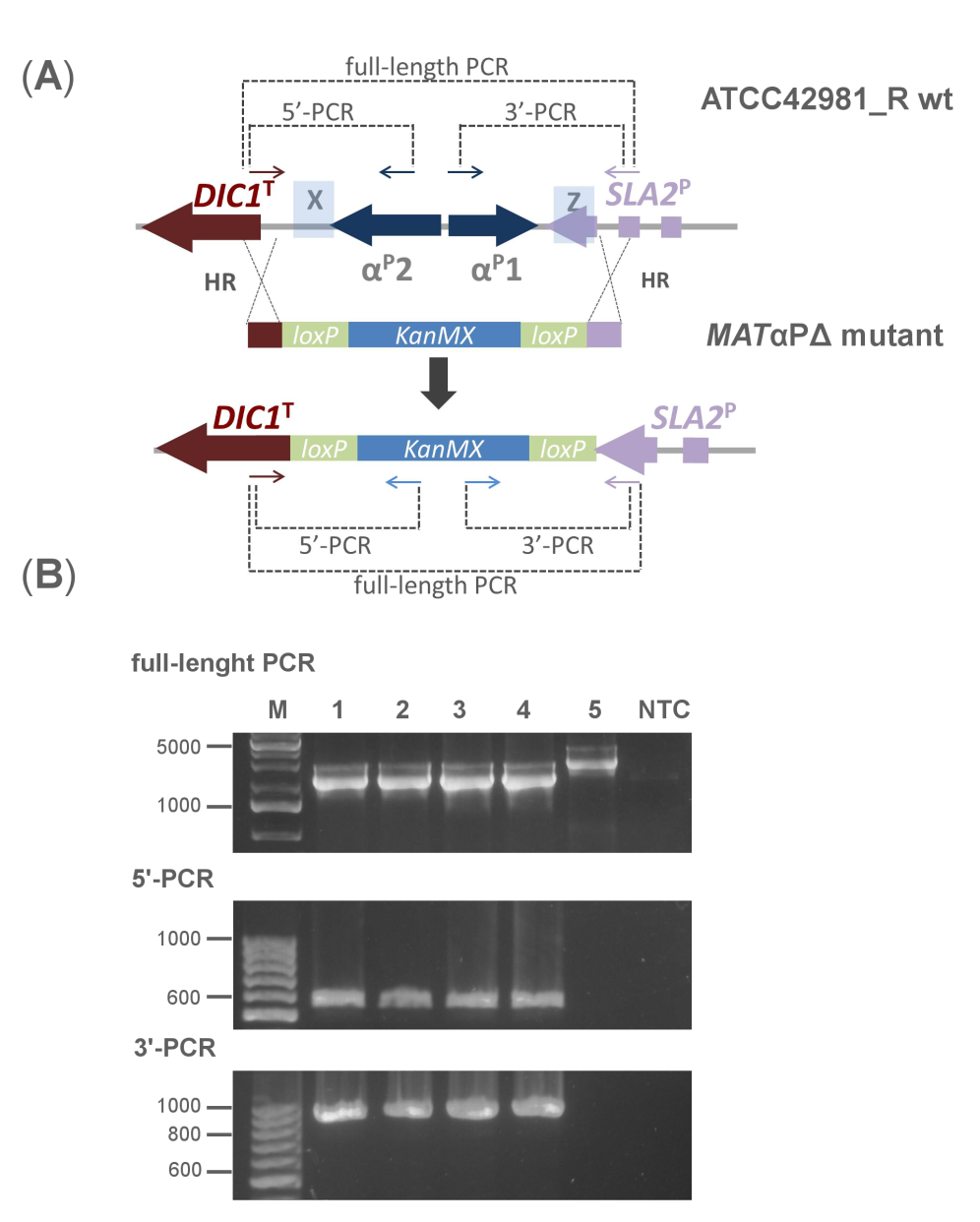
**

**Figure S1. Validation of *MAT*α^P^ deletion in ATCC42981_R.** Panel (**A**) outlines the diagnostic PCR strategy used to verify the integration of *loxP*-*kanMX*-*loxP* disruption cassette in the *DIC1*^T^-*MAT*α^P^-*SLA*2^P^ locus of ATCC42981_R genome. Full-length, 5’ and 3’-PCRs are shown. Flanking genes from T and P-subgenomes are marked with T and P superscripts, respectively. *MAT*α1 and *MAT*α2 genes from P-subgenome are indicated as α^P^1 and α^P^2. In panel (**B**) the *loxP*-*kanMX*-*loxP* module was integrated at the *DIC1*^T^-*MTL*α^P^-*SLA2*^P^ cassette in four ATCC42981_R clones, as demonstrated by size difference in full-length PCR products between *kanMX* (2,164 bp) and *MAT*α^P^ (2,889 bp) cassettes. The correct orientation of *loxP*-*kanMX*-*loxP* module was confirmed by the 5’ and 3’ PCRs. Numbers from 1 to 5 indicate *MAT*α^P^Δ clone_6, _65, _74, and _177, and ATCC42981_R, respectively (Table 1), while M represents molecular weight marker GeneRuler 100 bp or 1 Kb Plus DNA Ladders (Thermo Scientific, Waltham, MA). Abbreviations: wt, wild type; HR, homologous recombination; *kanMX*, kanamycin resistance gene; NTC, no template control.


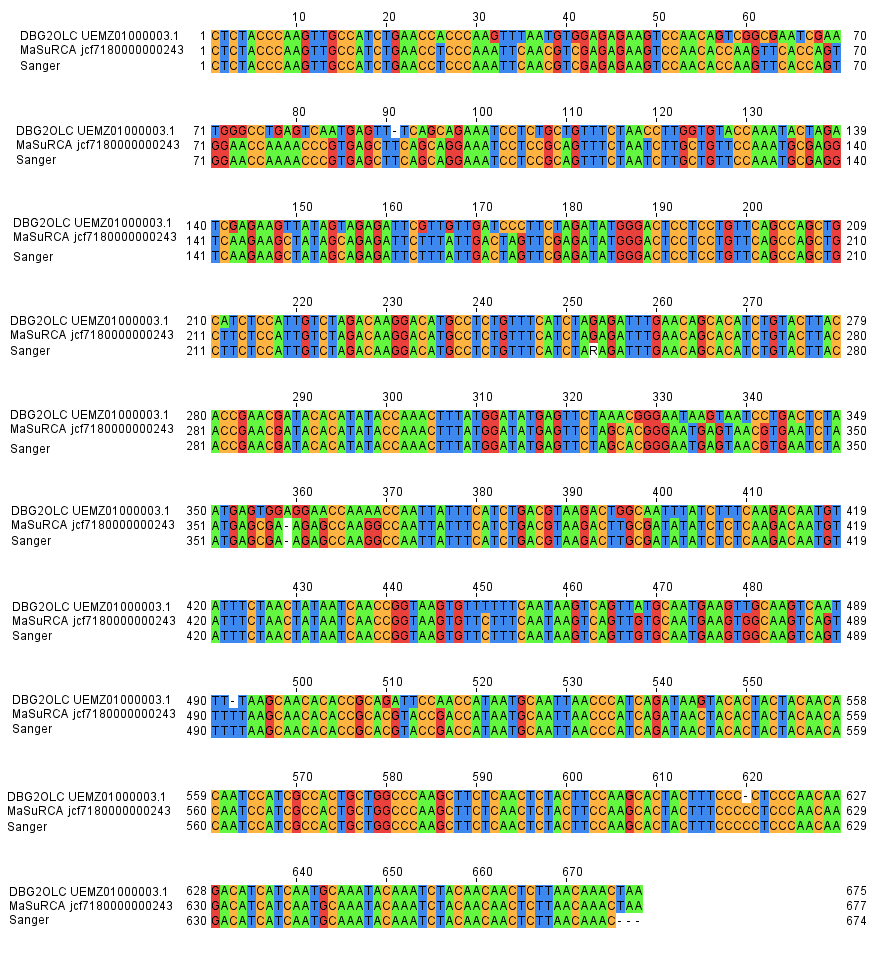


**Figure S2.** **Multiple sequence alignment of *SLA2* genes from DBG2OLC, MaSuRCA assemblies and Sanger sequences.** Alignment was carried out using Clustal Omega. Formatting of aligned sequences was done in Jalview alignment viewer. Residues in the alignment follow the default Clustal colour scheme of Jalview.


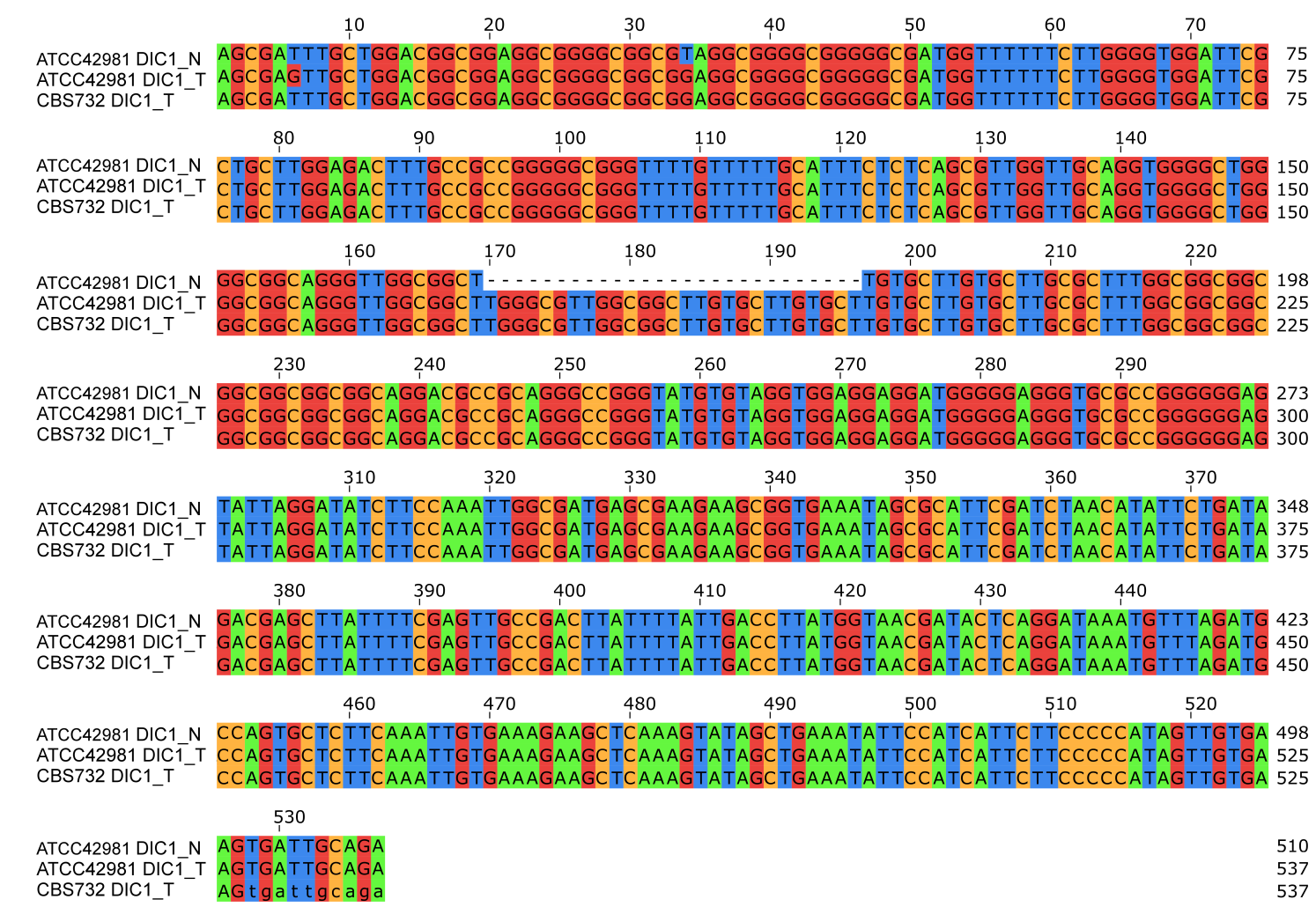


**Figure S3. Sequence alignment highlighting the 27 bp indel in X regions downstream the *DIC1^N^* and *DIC1^T^* gene variants.** X region from CBS732^T^ was used as reference. Sequences were retrieved from Sanger sequencing and aligned using Clustal Omega. Formatting of aligned sequences was done in Jalview alignment viewer. Residues in the alignment follow the default Clustal colour scheme of Jalview.


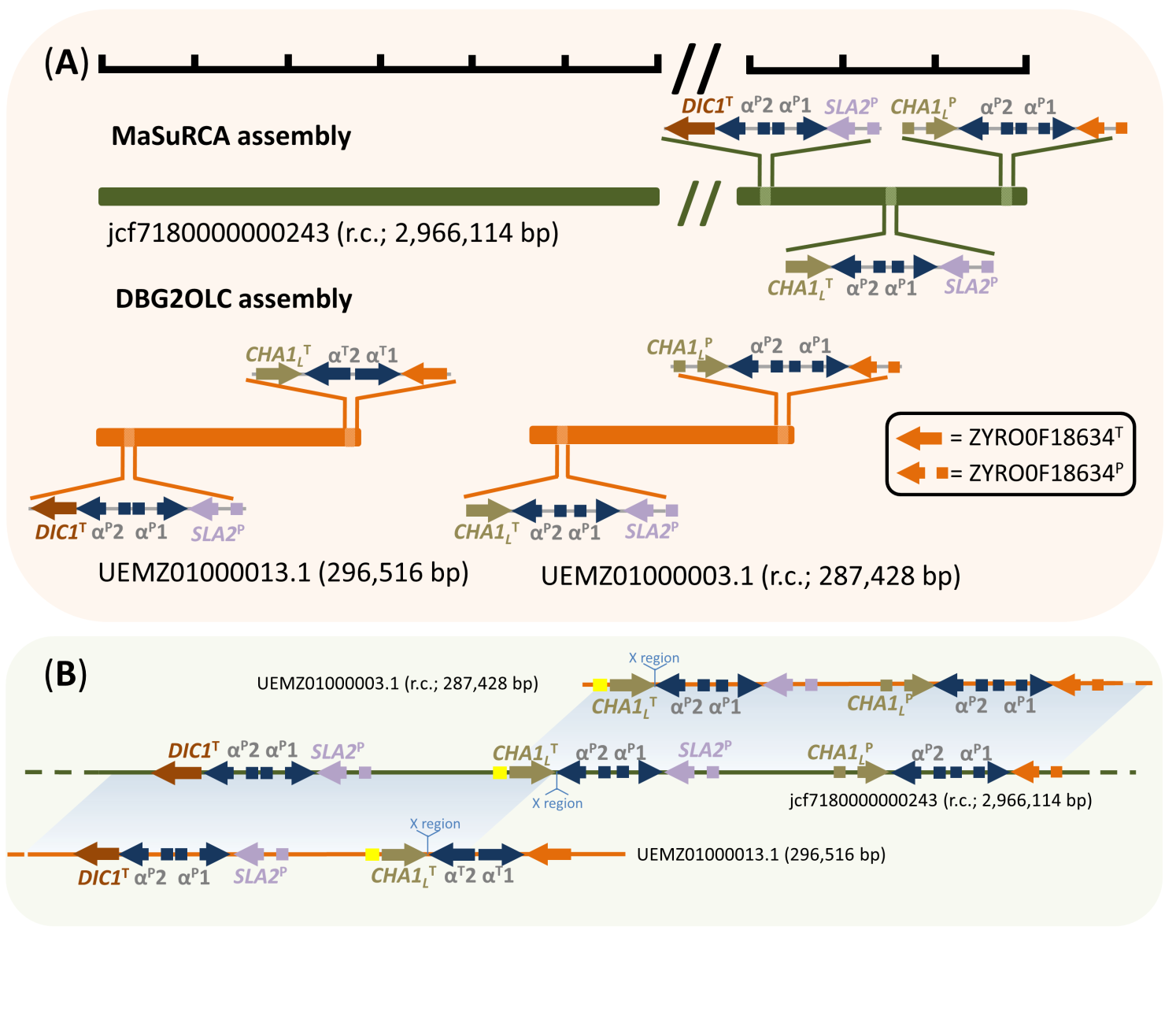


**Figure S4.** **Cartoon illustration of main differences between MaSuRCA and DBG2OLC assemblies in *MTL*α loci–containing scaffolds**. In panel (**A**) black horizontal bar represents size scale with tick marks for every 100 Kbp. MaSuRCA and DBG2OLC scaffolds are represented as green and orange rectangles, respectively. Synteny representation around *MTL*α loci omits X and Z regions for brevity. T and P variants are depicted as filled and dot arrows and marked with T and P superscripts, respectively. Panel (**B**) details collinear sets of homologous regions between the jcf180000000243 (r.c) MaSuRCA scaffold and either UEMZ01000013.1 or UEMZ01000003.1 (r.c) DBG2OLC scaffolds. Gene size and distance are not in scale. The yellow rectangle represents the starting point of scaffold synteny. Abbreviation: r.c., reverse complement.

**
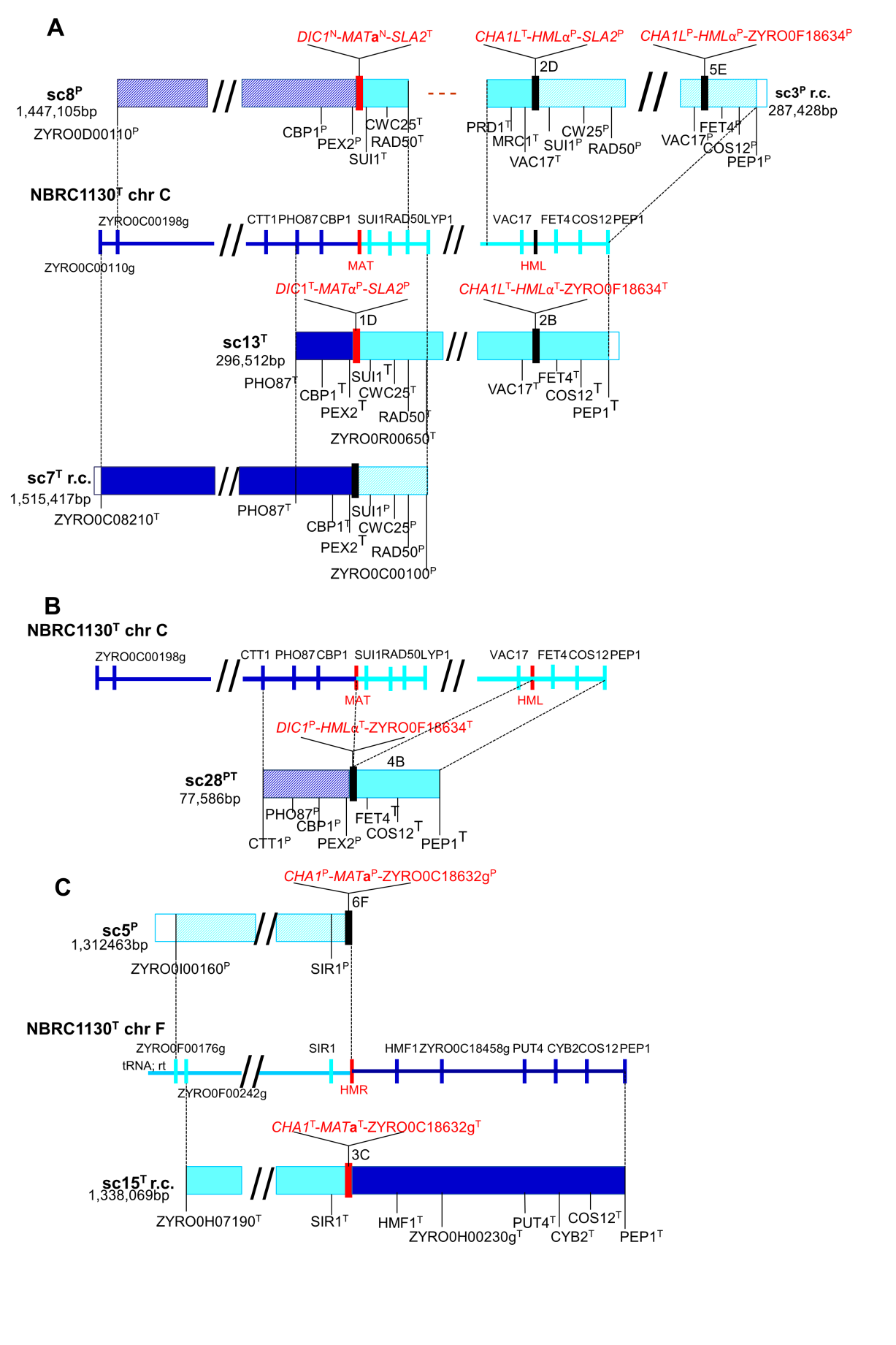
**

**Figure S5. Cartoon detailing gene order and synteny blocks around *MTL* loci in ATCC42981_R**. Rectangles represent regions in ATCC42981_R scaffolds that are collinear with a region of the reference strain NBRC1130^T^ ancestral genome. Blue and light blue colours correspond to regions that in CBS732^T^ genome are on chromosomes C and F, respectively. Solid rectangles and rectangles with diagonals represent T- and P-sequences, respectively. Scaffold (sc) numbers referred to the DBG2OLC genome assembly deposited in European Nucleotide Archive under accession number PRJEB26771 (Bizzarri et al., 2018); for simplicity the last number of ENA code marked each scaffold (*i.e*. UEMZ01000028.1 in short sc28). Genes from T and P-subgenomes are marked with T and P superscripts, respectively, while *DIC1* and *MAT***a**2 new variants with N superscript. Scaffolds are not in scale.
